# Implant Composed of Demineralized Bone and Mesenchymal Stem Cells Genetically Modified with AdBMP2/AdBMP7 for the Regeneration of Bone Fractures in* Ovis aries*

**DOI:** 10.1155/2016/7403890

**Published:** 2016-10-13

**Authors:** Adelina A. Hernandez-Hurtado, Gissela Borrego-Soto, Ivan A. Marino-Martinez, Jorge Lara-Arias, Viktor J. Romero-Diaz, Adalberto Abrego-Guerra, Jose F. Vilchez-Cavazos, Guillermo Elizondo-Riojas, Herminia G. Martinez-Rodriguez, Marcela A. Espinoza-Juarez, Gloria C. Lopez-Romero, Alejandro Robles-Zamora, Oscar F. Mendoza Lemus, Rocio Ortiz-Lopez, Augusto Rojas-Martinez

**Affiliations:** ^1^Departmento de Bioquímica y Medicina Molecular, Facultad de Medicina, Universidad Autónoma de Nuevo León, Monterrey, Mexico; ^2^Centro de Investigación y Desarrollo en Ciencias de la Salud, Universidad Autónoma de Nuevo León, Monterrey, Mexico; ^3^Banco de Huesos y Tejidos, Hospital Universitario, Universidad Autónoma de Nuevo León, Monterrey, Mexico; ^4^Departmento de Morfología, Facultad de Medicina, Universidad Autónoma de Nuevo León, Monterrey, Mexico; ^5^Facultad de Medicina Veterinaria y Zootecnia, Universidad Autónoma de Nuevo León, Monterrey, Mexico; ^6^Servicio de Ortopedia y Traumatología, Hospital Universitario, Universidad Autónoma de Nuevo León, Monterrey, Mexico; ^7^Servicio de Radiología e Imagen, Hospital Universitario, Universidad Autónoma de Nuevo León, Monterrey, Mexico

## Abstract

Adipose-derived mesenchymal stem cells (ADMSCs) are inducible to an osteogenic phenotype by the bone morphogenetic proteins (BMPs). This facilitates the generation of implants for bone tissue regeneration. This study evaluated the* in vitro* osteogenic differentiation of ADMSCs transduced individually and in combination with adenoviral vectors expressing BMP2 and BMP7. Moreover, the effectiveness of the implant containing ADMSCs transduced with the adenoviral vectors AdBMP2/AdBMP7 and embedded in demineralized bone matrix (DBM) was tested in a model of tibial fracture in sheep. This graft was compared to ewes implanted with untransduced ADMSCs embedded in the same matrix and with injured but untreated animals.* In vivo* results showed accelerated osteogenesis in the group treated with the AdBMP2/AdBMP7 transduced ADMSC graft, which also showed improved restoration of the normal bone morphology.

## 1. Introduction

Mesenchymal stem cells (MSCs) are adult cells with fibroblastoid morphology and ability to differentiate into multiple tissues including bone, fat, muscle, and cartilage [1]. MSCs are identified by the positive expression of* CD13*,* CD73*,* CD90,* and* CD105* genes, whereas they are negative for expression of hematopoietic markers such as CD34 and CD45 [2–4].

MSCs from adipose tissue are easily accessible and yield up to 5,000 fibroblast forming units (CFU-F) per gram of adipose tissue, in comparison to around 100–1000 CFU-F per milliliter from bone marrow [5, 6]. Its high proliferation rate facilitates expanding ADMSCs in the laboratory for therapeutic purposes. Furthermore, it has been shown that these cells have four properties that could be useful in cell therapy: angiogenicity, osteogenicity, immunomodulation, and promotion of tissue remodeling [7].

ADMSCs of different species can be induced to osteogenic differentiation by stimulation with some members of the bone morphogenetic protein family (BMPs) [8–10]. Although the mechanism is still unclear, multiple BMPs promote osteogenic differentiation of ADMSCs, mostly through the SMAD and the noncanonical Wnt mediated Wnt5a signaling pathways [11]. This osteogenic differentiation can be achieved by stimulation with homodimeric or heterodimeric combinations of BMP ligands. Some reports suggest that BMP2/BMP7 or BMP2/BMP9 combinations are more effective in inducing osteogenesis in MSCs [12–14].

Most preclinical trials for osteogenic induction using MSCs and BMPs have been tested in rodents and other small animals [15–18]. Preclinical trials in large animals are necessary to obtain morphological and biomechanical information of implants based on MSCs that try to repair bone defects in large mammals, particularly in bones supporting body weight such as leg bones [19–21]. In addition, some reports reveal differences in osteogenic potential between species [22]. Although preclinical data of implants for bone regeneration employing MSCs are increasing, data generated from large mammals, such as sheep, are needed for scaling and translating technologies for clinical trials.

Our team previously generated an implant for bone regeneration in a canine mandibular distraction model. The implant was constituted by bone marrow MSCs transduced with BMP2 and embedded in DBM [23]. For the present report, the challenge was to repair a major lesion in the tibia of a sheep model. This bone is involved in supporting body weight at rest and during the movement of this large mammal. To assess the quality of newly formed bone, physical and histological evaluations were performed to test this implant, by studying the speed of the osteogenic regeneration, the quality of the healed tissues, and the morphology of the regenerated bones. The study group treated with BMP2/BMP7 transduced ADMSCs was compared with controls including a group treated with an implant of nontransduced ADMSCs and with a group of injured but untreated sheep.

## 2. Materials and Methods

This study was approved by the Ethics Committee of the University Hospital of the Universidad Autonoma de Nuevo Leon (UANL) with approval number BI12-003. Care of the animal used during experimental protocols was conducted according to the Mexican Official Standard for the handling of laboratory animals (NOM-062-ZOO-1999) within the premises of the School of Veterinary Medicine of the UANL.

### 2.1. Generation of Adenoviral Vectors

Adenoviral vectors AdBMP7 and AdBMP9 were constructed using the AdEasy vector system (Agilent Technologies, Santa Clara, CA) according to the methodology by Luo et al. [24]. AdBMP7 and AdBMP9 are first generation serotype 5 adenoviruses (ΔE1, ΔE3) that are transgene carriers of the human BMP7 and BMP9 proteins, respectively, directed by the cytomegalovirus (CMV) early promoter. AdBMP2 is a previously described [25] adenoviral vector carrying the BMP2 protein, kindly donated by Dr. Cristopher Evans. Recombinant adenoviruses were amplified in HEK293 cells, purified with cesium chloride gradients and dialyzed in buffer consisting of 10 mM Tris-HCl (pH 8.0), 140 mM NaCl, 1 mM MgCl_2_, and 10% glycerol. Viral titration was performed by determining the optical density at 260 nm and by lytic plaques forming units according to the AdEasy vector system manual (Agilent Technologies).

### 2.2. Isolation and Characterization of Adipose Tissue ADMSCs

ADMSCs were isolated from biopsies of approximately 4 grams of sternal adipose tissue from sheep with a weight of 20 kg. Biopsies were transported at 4°C in phosphate buffer (PBS) supplemented with antibiotic/antimycotic and processed within the first 2 hours after lipectomy. Adipose tissue was excised and digested with type I collagenase 0.1% for 30 minutes at 37°C under constant stirring. After enzymatic digestion, it was centrifuged at 1,000 ×g for 5 minutes to form a cell pellet. The supernatant was discarded and 3 washes with PBS were performed to remove the fat phase and tissue debris. The cell pellet was resuspended in Dulbecco's Modified Eagle's medium (DMEM) (Invitrogen) supplemented with 1x glutamine (Invitrogen), 10% fetal bovine serum (FBS) (Invitrogen), and 1x antibiotic/antimycotic (GIBCO). Cells were incubated at 37°C with 5% CO_2_ for 3 days. Nonadherent cells were removed with the spent medium and medium changes were performed periodically until an 80% confluence.

For immunophenotyping 10^6^ cells per animal were used using anti-CD271-PE (Miltenyi Biotec), anti-MSCA-1 (W8B2)-APC (Miltenyi Biotec), and anti-CD45-FITC (Beckman Coulter) antibodies. CyAn ADP Analyzer (Beckman Coulter) cytometry was used for measurements. In addition to immunophenotyping, expression of the markers CD34, CD45, CD116, CD73, and GAPDH was determined by qPCR (quantitative real-time PCR) as an internal control using the primers listed in Supplementary Table S1 in Supplementary Material available online at http://dx.doi.org/10.1155/2016/7403890. For this, RNA extraction of total ADMSCs third passage with TRIzol® (Invitrogen) was performed following the manufacturer's instructions and cDNA was synthesized using SuperScript™ III First-Strand Synthesis SuperMix (Invitrogen), SYBR® GreenER™ qPCR SuperMix (Invitrogen), and they were analyzed by the 2^−ΔΔCt^ method. cDNA, 100 ng, was used for each reaction.

### 2.3. Differentiation of ADMSCs* In Vitro*

We seeded 90,000 cells/well in 6-well plaques and incubated these overnight at 37°C with 5% CO_2_. Afterwards, ADMSCs were transduced with AdBMP2, AdBMP7, AdBMP9 (MOI = 100) and AdBMP2/AdBMP7 and AdBMP2/AdBMP9 (MOI = 50/50) combinations for 3 hours; subsequently 3 washes with PBS were performed. A positive control in which cells were stimulated with osteogenic medium (Advanced DMEM 10% FBS, 50 *μ*M ascorbic acid, 10 mM *β*-glycerophosphate, and 100 mM dexamethasone) and a negative control of unstimulated cells were included. All experiments were performed in triplicate.

### 2.4. qPCR to Assess Osteogenic Differentiation

Osteogenic differentiation was assessed by osteocalcin (OC) and collagen type I (Col I) expression on days 8, 16, and 32 after induction using qPCR with the primers listed in Supplementary Table S1. Expression of the GAPDH gene was measured as a gene normalizer. The reactions were performed as described above. The values registered for the unstimulated cells group (negative control) were considered baseline levels of expression and these were subtracted from the values of the other two groups.

### 2.5. Histological Staining

Histological analyses were performed on days 1 and 32 after induction of ADMSCs transduced with individual and combined adenoviral vectors and positive and negative controls as mentioned above. Masson's trichrome stain was used to observe cell morphology and collagen deposits, and von Kossa staining was used to observe calcium deposits. The production of type I and type II collagen was evaluated by immunohistochemistry (IHC). Slides were observed under a Nikon Model E600 microscope.

### 2.6. ELISA

Production of BMP2 and BMP7 proteins was verified by ELISA in ADMSCs transduced with AdBMP2, AdBMP7, the combination AdBMP2/AdBMP7, and the positive and negative controls on days 8, 16, and 32 after induction, using the commercial kits Quantikine-ELISA Human BMP2 and Quantikine-ELISA Human BMP7 (R&D Systems).

### 2.7. Obtaining Demineralized Bone Matrix (DBM)

The DBM was obtained from sheep cancellous bone. The bones were ground in a special mill for bone. Traces of blood and fat were completely eliminated with a solution of 3% H_2_O_2_ and distilled, hot, sterile water. Subsequently, demineralization of the bone was conducted with 0.6 N HCl solution for 24 hours. Washes with distilled and sterile water were performed and the solution neutralized with a phosphate buffer at pH 7.0. The ground and demineralized bone was lyophilized to −0.070 mbar at −45°C for 24 hours and subsequently sterilized with gamma radiation.

### 2.8. Bone Regeneration Assays* In Vivo*

Twenty-one Pelibuey ewes of approximately 6 months of age and approximately 20 kg were used. Sheep were randomly assigned to three experimental groups made up of seven sheep each: Group I, control without an implant; Group II, implant with a DBM seeded with ADMSCs without transduction; Group III, implant with a DBM and ADMSCs transduced with AdBMP2/AdBMP7. The sheep were subjected to a first surgery to obtain adipose tissue, which was processed for the isolation of ADMSCs by enzymatic digestion with collagenase I, as described above. ADMSCs were grown to 10^6^ cells per animal.

#### 2.8.1. Generation and Grafting of Implants

Ten million ADMSCs obtained from adipose tissue for the generation of the implants were used; these were embedded in 1 cm^3^ of DBM in 24-well plates. For Group III, ADMSCs were previously transduced with the vectors AdBMP2/AdBMP7 for 3 hours and then 3 washes with PBS were performed as described for the differentiation* in vitro*. The implants were transported on ice under sterile conditions immediately to the School of Veterinary Medicine and Animal Husbandry where the surgical procedure was performed. Surgery consisted of a distraction of the tibia under general anesthesia with a first step of sedation with ketamine (1 mL/10 kg of weight) and xylazine (0.5 mL/kg of weight). Later, the anesthetic 5% isoflurane was administered via an intratracheal tube and administration of the anesthetic was continued at 1-2% throughout the surgery with oxygen. Aseptically and with the animal in lateral decubitus, the medial side of the posterior tibial shaft was approached. The periosteum in the same area was removed, the center of the tibial shaft was identified, and a block of bone of 1 cm^3^ was removed with the aid of a hand saw. One 3.5 mm dynamic compression plate (DCP) was placed, and a bone distraction of 10 mm was verified. The 3.5 mm DCP was fixed with 4 proximal and 4 distal osteotomy screws. Closure was performed with continuous 2-0 vicryl suture. Postoperative recovery was carried out in individual metabolic cages. During the next 7 days postsurgically, 1-2 mL/40 kg/24 h of Ceftiofur sodium (40 mg/mL) and 1-2 mL/50 kg/24 h of Flunixin (50 mg/mL) were administered intramuscularly; also, daily cleaning of the wound was carried out until healing with an antiseptic povidone-iodine solution and 10% Aluspray® (Vetoquinol, France).

#### 2.8.2. Radiographic Analysis and 3D Tomography

Immediately postoperative, anteroposterior, and lateral X-rays were performed to confirm the space between the proximal and distal ends of the tibia. Each experimental group was given radiographic followup at weeks 4, 7, and 10. Five radiographic images of each animal at different observation times were obtained. 3D computerized tomography (CT) scan was performed in postmortem specimens of the injured bones from each animal. The bone segments were arrayed in the experimental groups abs immobilized in vertical position on a cardboard surface and were analyzed with a General Electric Light Speed VCT™ system using the following protocol: 100.0 kV, 100 mA, and 0.6 mm. This study was performed to study bone shapes, transversal structure and density, and bone regeneration.

#### 2.8.3. Specimen Collection and Evaluation of Osteogenesis

Ten weeks after surgery, the sheep were euthanized with an intravenous overdose of phenobarbital-KCl and implants were retrieved for a postmortem neoformed bone assessment with histological staining of the fragment of the injured area of the sheep from each group. Before fixation, bone specimens were analyzed by CT scan as described before. For histological staining, implant areas were removed and fixed with formalin and glutaraldehyde for 7 days and decalcified with formic acid 10% for two weeks and 2 N HCl for 24 h. They were then dehydrated in alcohol and included in paraffin. Five-micron thick sections were made for staining with H&E and Masson's trichrome to observe morphology/cell repopulation and bone matrix synthesis, respectively. IHC was also performed for detection of collagen type I, collagen type II, and collagen type X, for which a pretreatment in buffer citrate pH = 6.0 was done, which consisted of preheating at 65°C and then incubation at 85°C for 20 minutes. Anti-collagen type I (rabbit polyclonal, dilution 1 : 300, cat. # ab34710), anti-collagen type II (rabbit polyclonal, dilution 1 : 500, cat. # ab34712), and anti-collagen type X (rabbit polyclonal, dilution 1 : 500, cat. # ab58632) antibodies from Abcam™ were used. Sections were incubated with antibodies for 15 mins at room temperature and detected using the Mouse & Rabbit Specific HRP/DAB Detection IHC Kit, also from Abcam. Sections were counterstained with hematoxylin.

#### 2.8.4. Biosafety and Toxicity Studies

Biosafety tests were conducted to evaluate the possible effect of adenoviral vectors on animals (particularly, inflammatory liver injury). Liver function tests and a blood count of each animal was performed one day before taking adipose tissue and 20 days after implant surgery. For each of the sheep, two blood samples from the jugular vein before and after the procedure were drawn.

### 2.9. Statistical Analysis

Expression data were analyzed to find statistically significant differences between the values of each experimental group using Student's *t*-test. SigmaPlot v11 (Systat Software) was used. The data are presented as mean ± standard deviation. The data obtained from ELISA tests were analyzed by the ANOVA test. A *P* value < 0.05 was considered significant.

## 3. Results

### 3.1. Characterization of ADMSCs

The cells isolated from adipose tissue showed fibroblastoid morphology and the mesenchymal phenotype was confirmed by positivity for the surface markers CD271 and MSCA-1 (85% of the cell population for both markers) and negative for CD45. qPCR demonstrated expression of CD166 and CD73 characteristic of ADMSCs and absence of CD45 and CD34, characteristic markers of hematopoietic cell lines.

### 3.2. *In Vitro* Differentiation Assays

**Figure 1:**
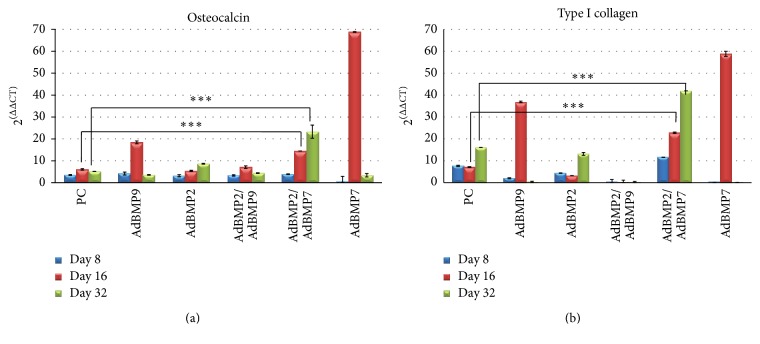
Relative expression analysis of (a) osteocalcin and (b) type I collagen by qPCR of MSCs transduced with AdBMP-2, AdBMP7, AdBMP9, and the combinations AdBMP2/AdBMP7 and AdBMP2/AdBMP9 on days 8, 16, and 32 after induction. The combination AdBMP2/AdBMP7 shows higher levels of osteocalcin and osteopontin and these were maintained for 32 days. ^*∗∗∗*^significance level *P* < 0.05.

Relative expression analysis of osteocalcin I and type I collagen mRNA was measured at different days of observation (days 8, 16, and 32) in the following groups: a positive control of ADMSCs cultured in osteogenic medium, ADMSCs singled transduced with AdBMP2, AdBMP7, AdBMP9, and combined transductions with AdBMP2/AdBMP7 and AdBMP2/AdBMP9. This study showed significantly increased levels of tested messengers in the group treated with the AdBMP2/AdBMP7 combination (Figures 1(a) and 1(b)). The effect in the group treated with AdBMP2 also showed a sustained, but less intense effect, similar to that observed in the control group for osteocalcin, and low levels of transcripts for collagen I. Although ADMSCs transduced with AdBMP7 had the highest expression levels of osteocalcin I and collagen type I, these were not sustained until day 32, as what also occurred with AdBMP9 and the AdBMP2/AdBMP9 combination. Additionally, cell death was observed in the cultures treated with AdBMP9 alone and in combination at day 32 of incubation. For this reason it was not possible to determine the production of proteins in subsequent assays.

**Figure 2:**
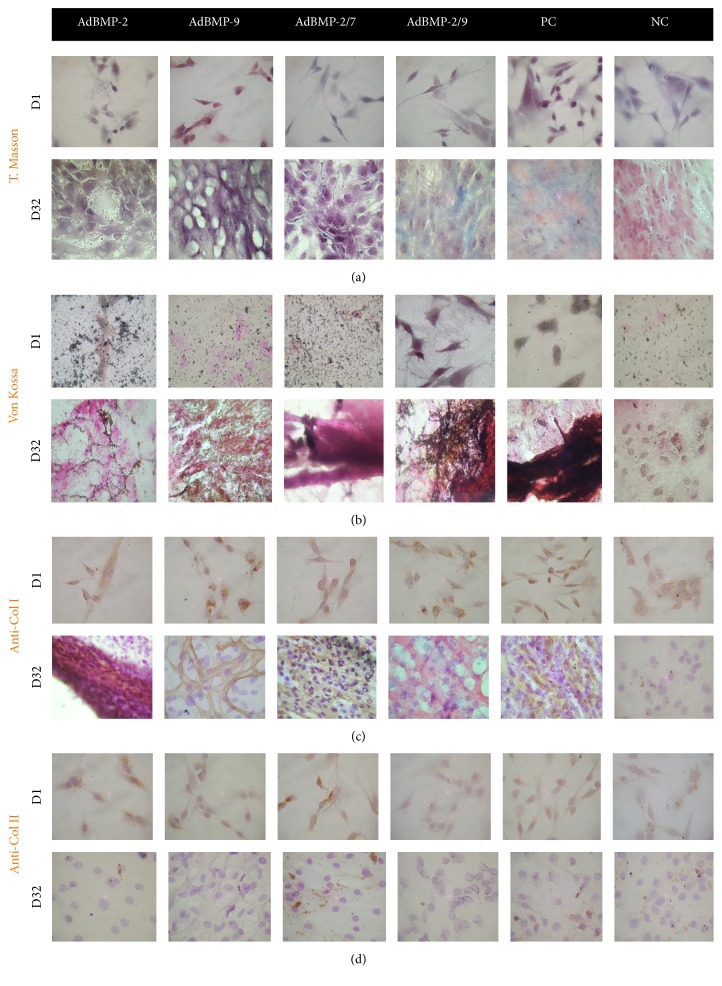
Histological staining at days 1 and 32 of culture. Masson's trichrome for the identification of collagen and amorphous material; (b) von Kossa for the identification of calcifications; (c) IHC for collagen I; (d) IHC for collagen II. PC: positive control; NC: negative control. Micrographs with 10x amplification.

Special stains, Masson's trichrome and von Kossa, were used to identify collagen and calcium deposits, respectively, in the experimental groups. Masson's trichrome showed the presence of collagen matrix on day 32 in ADMSCs transduced with the AdBMP2/AdBMP7 combination with a discrete blue staining of collagen mesh. However, the AdBMP2/AdBMP9 combination showed a higher proportion of collagen, resembling the staining of the positive control group (Figure 2(a)). The von Kossa stain identified scattered calcium deposits on the slides at day 1 after induction. The deposits at day 32 were mostly concentrated in a colocalization with the cell population; more deposits were observed with the combinations AdBMP2/AdBMP7 and AdBMP2/AdBMP9, with the combination AdBMP2/AdBMP7 being most similar to the positive control in cell organization and intensity of staining (Figure 2(b)). The presence of type I collagen by IHC was well established in the AdBMP2, AdBMP9 groups and with the combination AdBMP2/AdBMP7, with the latter group presenting a more uniform distribution of collagen type I fibers between cells (Figure 2(c)). Collagen type II was detected slightly in most cells at day 1 of culture and was absent in all groups at day 32, except for the combination AdBMP2/AdBMP7 (Figure 2(d)).

**Figure 3:**
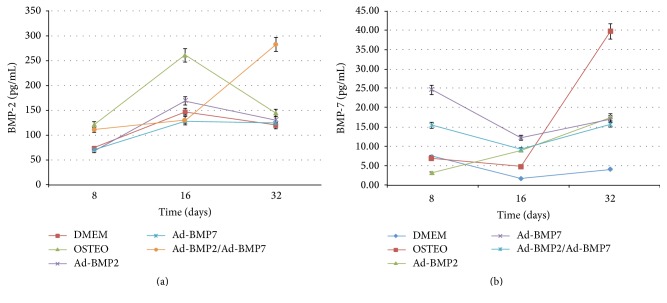
ELISA. (a) Analysis of the protein BMP2 and (b) BMP7 by ELISA of MSCs transduced with the vectors AdBMP2, AdBMP7, the combination AdBMP2/AdBMP7, and the positive (OSTEO) and negative (DMEM) controls on days 8, 16, and 32 after induction.

Levels of BMP2 and BMP7 proteins measured by ELISA showed that expression of BMP2 was sustained up to day 32 in the group transduced with the combination AdBMP2/AdBMP7, with this expression being different from the rest of the groups in the same observation period (*P* < 0.05) (Figure 3(a)). Conversely, expression of BMP7 at day 32 was higher in the positive control; however, at days 8 and 16, the expression of BMP7 in the ADMSCs groups transduced with AdBMP7 and the AdBMP2/AdBMP7 combination showed greater expression with their levels being maintained up to day 32 (Figure 3(b)). Different statistical differences were established for the BMP2 and BMP7 expression levels between groups in each of the observation points (ANOVA *P* < 0.05).

### 3.3. Bone Regeneration Assays* In Vivo*

**Figure 4:**
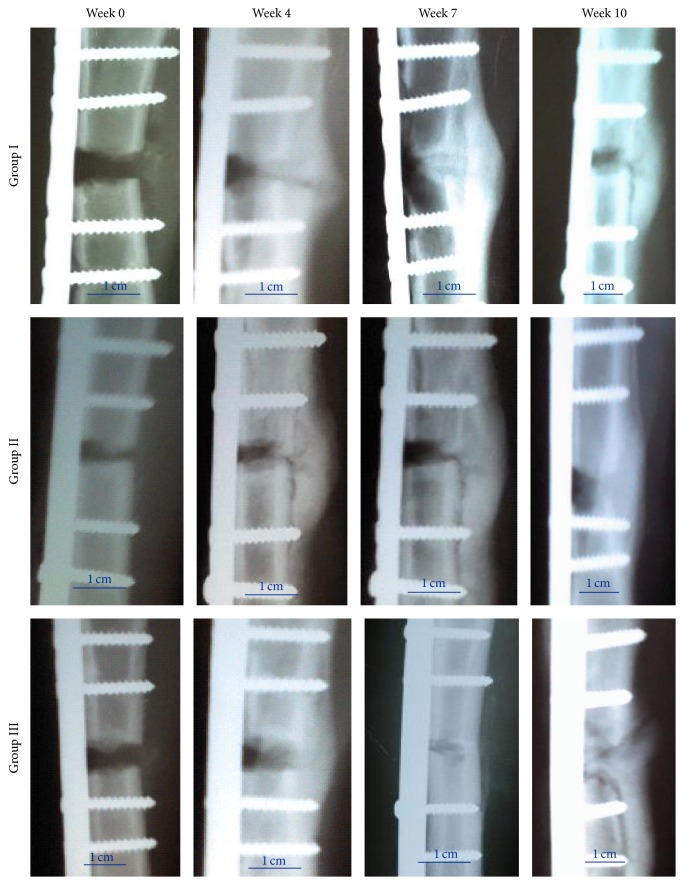
Radiographic followup at 0 to 10 weeks of treatment groups: Group I, control without an implant; Group II, implant with a DBM seeded with MSCs without transduction; and Group III, implant with a DBM and MSCs transduced with AdBMP2/AdBMP7. Scale bar: 1 cm.

Radiographic followup at 10 weeks showed a faster closure of the lesions in Group III (Figure 4). The lesion in this group was completely filled at week 7 after implant. In contrast, bone distractions in the Groups I and II were not fully filled at week 10 of followup. Radiographic images also showed that bone formation in control groups was altered, causing a bone deformation in the area of the lesion, while in Group III, the X-ray showed that the lesion was uniformly filled, resulting in a bone appearance and consistency similar to that of a healthy bone.

**Figure 5:**
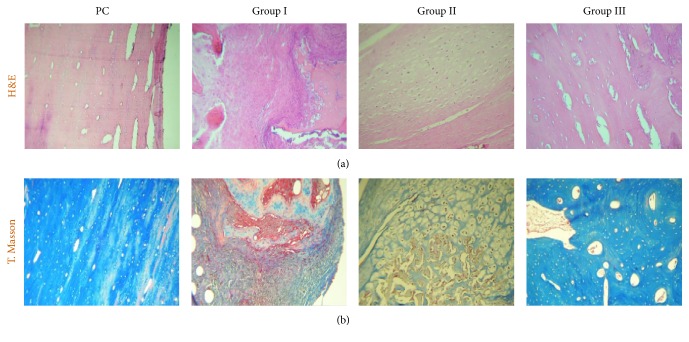
Postmortem histological staining. (a) H&E; (b) Masson's trichrome. PC: positive control. Micrographs with 10x amplification.

Postmortem H&E histological comparisons among the groups, including healthy tibia as a positive control (PC), showed that bones in Group III have preserved periosteum and the cortex showed a compact and uniform bone matrix, consisting of osteocollagen, with immersed gaps of bone cells (osteocytes) and blood vessels in the lamellar bone architecture. In Group I, periosteal thickening was seen, and areas of dense fibrous tissue were observed in cortical and trabecular bone. In the medullary cavity, extended fibrosis with scarce cancellous bone, lack of hyaline cartilage, and abundant amorphous and fibrillar material was observed. Furthermore, lymphocyte, plasma cells, and macrophages infiltrates were also observed. Group II showed thickening of periosteum, while the bone cortex showed formation of highly vascularized fibrocollagenous zones. Small areas of hypertrophic hyaline cartilage were present in the medullary cavity of the lesion with large areas of fibrous tissue. In Group III, the periosteum appeared thin and slightly fibrous, with abundant stellate and fusiform differentiating cells. Bone cortex showed abundant cancellous tissue constituted by a uniform and compact matrix without cartilaginous tissue. The proportion of compact bone tissue was greater than in groups I and II (Figure 5(a)).

**Figure 6:**
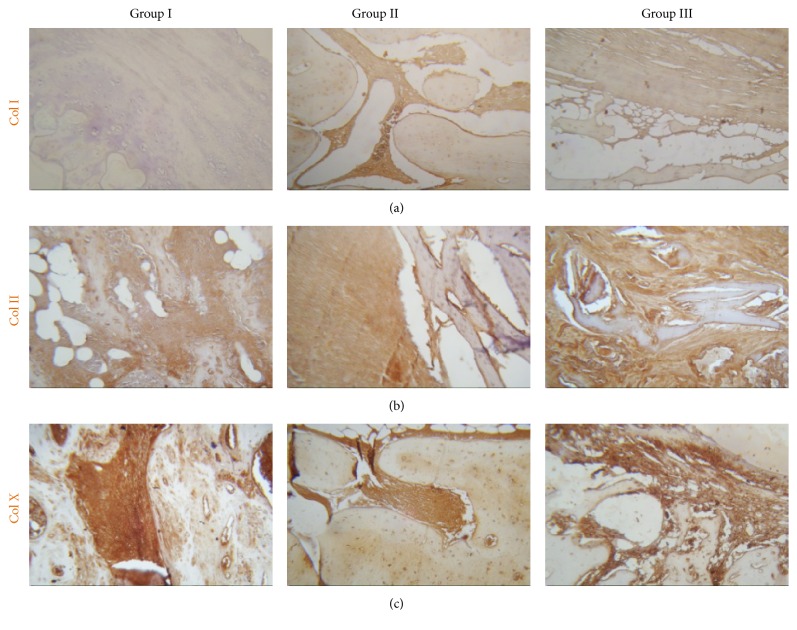
Postmortem IHC. (a) IHC for collagen I; (b) IHC for collagen II and (c) IHC for collagen X. Micrographs with 10x amplification.

The blue coloring of the collagen matrix by Masson's trichrome demonstrated that production of collagen matrix in Group III was higher when compared to the other groups, which showed fibrous tissue and amorphous material. The compact and uniform collagen matrix of Group III resembled the matrix of healthy tibia (Figure 5(b)). IHC studies showed type I collagen in all three groups (Figure 6(a)). Focal areas of hyaline cartilage showed weak staining for type II collagen in Group I (Figure 6(b)). Type X collagen was present in trabecular bone in all groups (Figure 6(c)).

### 3.4. 3D CT-Scan Analysis

**Figure 7:**
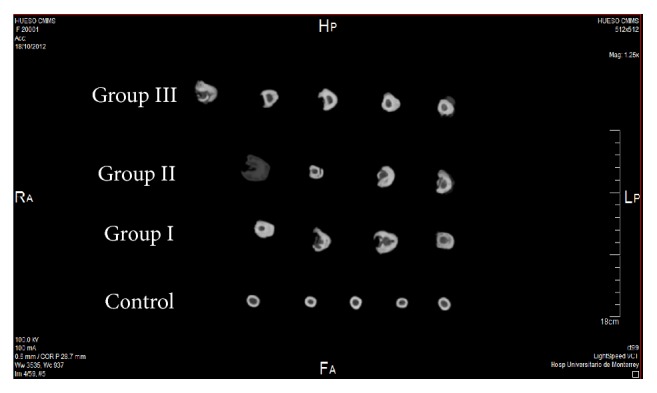
3D computerized tomography of a sagittal slice of sheep bones 10 weeks after surgery (postmortem) represented by group.

**Figure 8:**
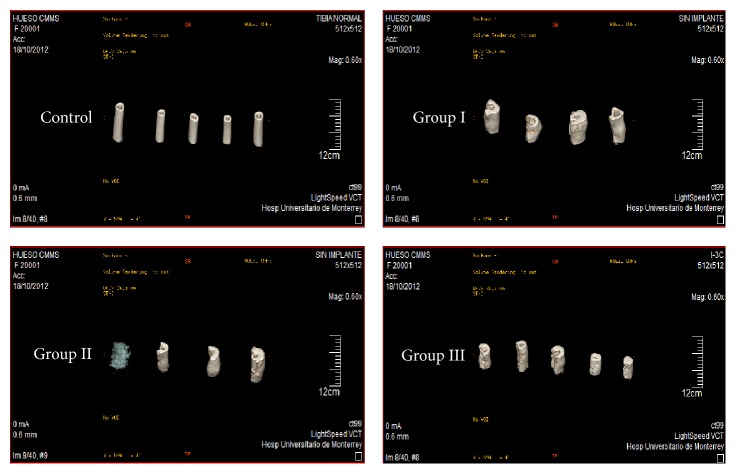
3D computerized tomography of the area of a lesion in each sheep by group 10 weeks after surgery (postmortem).

Sagittal sections of the treated tibia from each sheep per group were performed and noninjured tibias were used as healthy controls (Figure 7). In Groups I and II, bones were totally deformed by the formation of calluses in the area of the lesion without defining the cortex and medulla, while in Group III images that more closely resembled a healthy tibia they were seen. In Figure 8 the lesion area of each sheep is shown and it can be seen that, in Group III, bones are more uniform in appearance, size, and cortical density, similar to the control tibias.

### 3.5. Biosafety and Toxicity Studies

Table 1 shows the laboratory tests that were performed before and 20 days after surgery to assess the overall toxicity of adenoviral vectors used in Group III. It can be seen that the results were within normal values reported for this species, showing that no inflammatory processes or alterations in liver function tests demonstrate any adverse effects.

## 4. Discussion

This study analyzed the* in vitro* osteogenic potential of ovine ADMSCs transduced with AdBMP2, AdBMP7, and AdBMP9 alone and in the combinations AdBMP2/AdBMP7 and AdBMP2/AdBMP9. The aim was to determine the optimum components for the design of an implant for bone regeneration preclinical trials in a mammal model for a major bone lesion that allows thinking about future translation of this therapy into clinical trials.

*In vitro* gene expression studies demonstrated that the combination AdBMP2/AdBMP7 stimulates sustained expression of osteogenic markers until day 32 of observation. Histology of this group shows a production of a collagen matrix and suitable mineralization. IHC showed that collagen type I is the predominant protein in the collagen matrix formed by the ADMSCs stimulated with AdBMP2/AdBMP7. Although AdBMP7 alone stimulates increased expression of both osteogenic markers by day 16, these levels fall at day 32 in* in vitro* assays. Nonsustained expression of osteogenic markers in this group and the loss of adherence to the culture dish may suggest an altered osteogenic differentiation.

Zhu et al. evaluated the expression of osteogenic markers in cells A549, C2C12, and MC3T3-E1 transduced with the adenoviral vectors carrying BMP7, BMP2, and an adenovirus without transgene, alone and in combination. They found that, after 12 days of transduction, the combination BMP2 and BMP7 overexpressed alkaline phosphatase and osteocalcin 6 to 40 times more than treatment with BMP2 and BMP7 alone [26]. In addition, this combination significantly increased the rate of bone formation and spinal fusions in a mouse model [26, 27].

This study also demonstrates that BMP2 and BMP7 proteins are produced at high concentrations. BMP2 was expressed higher and more consistently, particularly in cells transduced with the combination Ad-BMP2/AdBMP7 at day 32 of incubation. Koh and colleagues, who used transduced fibroblasts with adenovirus carrying the genes of the BMP2 and BMP7 to regenerate craniofacial bone, reported similar results. These researchers also observed high alkaline phosphatase production with this combination [28]. Qing et al. studied bone regeneration using stem cells derived from rat adipose tissue transduced with lentivirus carriers of the BMP2 and BMP7 genes. They observed high alkaline phosphatase activity, calcium deposits, and high expression of the osteogenic markers osteocalcin and osteopontin after 14 days of incubation [29]. Currently, the recombinant proteins rhBMP2 and rhBMP7 have already been approved by the Food and Drug Administration (FDA) to be used in clinical trials [30]. Some reports of the use of rhBMP2 and rhBMP7 in bone regeneration trials in rodents show promising results that demonstrate ossification between weeks 6 and 8 after treatment [26, 27, 31–33].

In an ovine model of bone distraction, the osteogenic potential of rhBMP2 at a dose of 4 mg/day on days 3, 10, and 17 after distraction of the tibia was evaluated and it was observed that 3 days of treatment were sufficient to regenerate normal trabecular microarchitecture [34]. However, the production of recombinant proteins is still a costly process and treatment requires repeated applications, which increases the cost of therapy. Recently, a slow release method for rhBMP2 and rhBMP7 was tested in a fibrin hydrogel of hyaluronic acid for regeneration of spinal disc in goats in a period of 12 weeks; but no disc regeneration was observed in any of the study groups. The authors postulate that this could occur due to the low doses of BMPs, a short followup time, and/or insufficient release of the heterodimers rhBMP2/rhBMP7 [20].

Alternative techniques based on gene transduction using naked DNA or viral vectors for the expression of BMPs can be option for the development of self-stimulated implants that steadily express growth factors at the site of the lesion, in large and small mammals [29, 35–37]. However, the injection of AdBMP2 and Ad-BMP7 in the site of an osteotomy in 450 kg equines to induce osteogenesis after a 16-week evaluation did not show differences between the control and the transduced groups [38].

*In vivo* analysis of this study showed a satisfactory recovery of sheep in Group III. Radiologic studies showed that complete bone healing in this group was achieved in less time (7–10 weeks) unlike other experimental groups where consolidation was not achieved and bone deformation was observed. In the 3D CT scan, it was observed that, in Group III, shape, size, structure, and cortical density were more similar to the control group than to Groups I and II. Also, no liver or inflammatory disorders due to the use of adenoviral vectors were found. Dong and colleagues reported that complete bone healing was achieved using MSCs transduced with a recombinant adenovirus AdCbfa1a at 12 weeks after a lesion to the right radius in the front leg in rabbits [39]. In the canine model where the implant with MSCs transduced with AdBMP2/AdBMP7 was previously tested, radiographic followup showed complete healing in the treated jaw at week 6 after distraction [23]. These data indicate a marked reduction in bone healing time when genetically modified MSCs are used.

In summary, the discussed reports illustrate the technical difficulties that persist for the development of a promising implant, but the results of the present study support the generation of an optimal implant for bone regeneration in sheep.

### 4.1. Conclusions

The present study demonstrates the enhanced and sustained effect of the transduction of ovine ADMSCs with a combination of BMP2 and BMP7 that is suitable for the development of a bone regeneration implant to be tested in a sheep model with the purpose of advancing preclinical analyses for future clinical trials of bone regeneration.

## Supplementary Material

Table S1. Primers used in this study for qPCR analyses.

## Figures and Tables

**Table 1:** Toxicity studies related to the use of adenoviral vectors.

Analytical parameter	Reference range	Group I		Group II		Group III	
		Pre S	Post S	Pre S	Post S	Pre S	Post S
Leukocytes (*μ*L)	4000–12000	5507.5 ± 0.3	11086 ± 0.3	9748.3 ± 0.3	8375 ± 0.2	11251.7 ± 0.5	8758.3 ± 0.3
Hemoglobin (g/dL)	9–15	11.9 ± 0.2	9.72 ± 0.1	10.2 ± 0.2	10.3 ± 0.1	10.7 ± 0.1	10.1 ± 0.1
Platelets (thousands/*μ*L)	220–680	376.2 ± 0.5	832.2 ± 0.3	470.3 ± 0.2	779.3 ± 0.4	684.1 ± 0.4	720.8 ± 0.5
PT (g/dL)	6.0–7.9	6.36 ± 0.1	6.68 ± 0.1	6.1 ± 0.0	7.1 ± 0.1	6.8 ± 0.1	6.8 ± 0.1
Albumin (g/dL)	2.4–3.0	3.46 ± 0.1	2.9 ± 0.1	3.1 ± 0.1	3.1 ± 0.1	3.1 ± 0.1	3.2 ± 0.1
Globulin (g/dL)	3.5–5.7	2.86 ± 0.1	3.76 ± 0.2	3.05 ± 0.1	4.2 ± 0.3	3.7 ± 0.2	3.5 ± 0.2
AST (IU/L)	66–194	146.4 ± 0.4	174.6 ± 0.3	131.5 ± 0.3	140.3 ± 0.6	112.1 ± 0.1	104.3 ± 0.2
ALT (IU/L)	12–37	17.8 ± 0.3	27 ± 0.9	19 ± 0.5	16.7 ± 0.3	15 ± 0.6	16.5 ± 0.4
Total bilirubin (mg/dL)	0.0–1.0	0.108 ± 0.4	0.032 ± 0.4	0.1 ± 0.6	0.04 ± 1.1	0.06 ± 0.6	0.04 ± 0.8
Total bilirubin (mg/dL)	0.0–0.2	0.03 ± 0.2	0.03 ± 0.4	0.1 ± 0.8	0.1 ± 1.2	0.04 ± 1.0	0.1 ± 0.7
Indirect bilirubin (mg/dL)	0.0–1.0	0.076 ± 0.6	0.03 ± 0.4	0.1 ± 0.8	0.1 ± 1.2	0.04 ± 1.0	0.1 ± 0.7
Alkaline phosphatase (IU/L)	68–387	305.8 ± 0.5	134.4 ± 0.2	167.5 ± 0.6	188.5 ± 0.3	198 ± 0.4	211.3 ± 0.3
GGT (IU/L)	36–102	53.8 ± 0.2	51.8 ± 0.1	68.2 ± 0.3	73.7 ± 0.3	49.07 ± 0.2	58.0 ± 0.4

Pre S: presurgery; Post S: postsurgery.

## References

[1] Salehi H., Amirpour N., Niapour A., Razavi S. (2016). An overview of neural differentiation potential of human adipose derived stem cells. *Stem Cell Reviews and Reports*.

[2] Kasten P., Beyen I., Egermann M. (2008). Instant stem cell therapy: characterization and concentration of human mesenchymal stem cells in vitro. *European Cells and Materials*.

[3] Muñiz C., Teodosio C., Mayado A. (2015). Ex vivo identification and characterization of a population of CD13high CD105^+^ CD45^−^ mesenchymal stem cells in human bone marrow. *Stem Cell Research & Therapy*.

[4] Kobolak J., Dinnyes A., Memic A., Khademhosseini A., Mobasheri A. (2016). Mesenchymal stem cells: identification, phenotypic characterization, biological properties and potential for regenerative medicine through biomaterial micro-engineering of their niche. *Methods*.

[5] Romagnoli C., Brandi M. L. (2014). Adipose mesenchymal stem cells in the field of bone tissue engineering. *World Journal of Stem Cells*.

[6] Zhang X., Hirai M., Cantero S. (2011). Isolation and characterization of mesenchymal stem cells from human umbilical cord blood: reevaluation of critical factors for successful isolation and high ability to proliferate and differentiate to chondrocytes as compared to mesenchymal stem cells from bone marrow and adipose tissue. *Journal of Cellular Biochemistry*.

[7] Pill K., Hofmann S., Redl H., Holnthoner W. (2015). Vascularization mediated by mesenchymal stem cells from bone marrow and adipose tissue: a comparison. *Cell Regeneration*.

[8] Fu H. L., Diao Z. Y., Shao L., Yang D. P. (2014). BMP-2 promotes chondrogenesis of rat adipose-derived stem cells by using a lentiviral system. *Genetics and Molecular Research*.

[9] Lin Z., Wang J.-S., Lin L. (2014). Effects of BMP2 and VEGF165 on the osteogenic differentiation of rat bone marrow-derived mesenchymal stem cells. *Experimental and Therapeutic Medicine*.

[10] Taşli P. N., Aydin S., Yalvaç M. E., Şahin F. (2014). Bmp 2 and Bmp 7 induce odonto-and osteogenesis of human tooth germ stem cells. *Applied Biochemistry and Biotechnology*.

[11] Zhang X., Guo J., Zhou Y., Wu G. (2014). The roles of bone morphogenetic proteins and their signaling in the osteogenesis of adipose-derived stem cells. *Tissue Engineering Part B: Reviews*.

[12] Drevelle O., Daviau A., Lauzon M.-A., Faucheux N. (2013). Effect of BMP-2 and/or BMP-9 on preosteoblasts attached to polycaprolactone functionalized by adhesive peptides derived from bone sialoprotein. *Biomaterials*.

[13] Hankenson K. D., Gagne K., Shaughnessy M. (2015). Extracellular signaling molecules to promote fracture healing and bone regeneration. *Advanced Drug Delivery Reviews*.

[14] Khosla S., Westendorf J. J., Oursler M. J. (2008). Building bone to reverse osteoporosis and repair fractures. *The Journal of Clinical Investigation*.

[15] Jang Y. S., Choi C. H., Cho Y. B., Kang M.-K., Jang C. H. (2014). Recombinant human BMP-2 enhances osteogenesis of demineralized bone matrix in experimental mastoid obliteration. *Acta Oto-Laryngologica*.

[16] Visser R., Bodnarova K., Arrabal P. M., Cifuentes M., Becerra J. (2016). Combining bone morphogenetic proteins-2 and -6 has additive effects on osteoblastic differentiation in vitro and accelerates bone formation in vivo. *Journal of Biomedical Materials Research. Part A*.

[17] He X., Liu Y., Yuan X., Lu L. (2014). Enhanced healing of rat calvarial defects with MSCs loaded on BMP-2 releasing chitosan/alginate/hydroxyapatite scaffolds. *PLoS ONE*.

[18] Wang G. X., Hu L., Hu H. X., Zhang Z., Liu D. P. (2014). In vivo osteogenic activity of bone marrow stromal stem cells transfected with Ad-GFP-HBMP-2. *Genetics and Molecular Research*.

[19] Liu F., Ferreira E., Porter R. M. (2015). Rapid and reliable healing of critical size bone defects with genetically modified sheep muscle. *European Cells and Materials*.

[20] Peeters M., Detiger S. E., Karfeld-Sulzer L. S. (2015). BMP-2 and BMP-2/7 heterodimers conjugated to a fibrin-hyaluronic acid hydrogel in a large animal model of mild intervertebral disc degeneration. *BioResearch Open Access*.

[21] Geuze R. E., Theyse L. F. H., Kempen D. H. R. (2012). A differential effect of bone morphogenetic protein-2 and vascular endothelial growth factor release timing on osteogenesis at ectopic and orthotopic sites in a large-animal model. *Tissue Engineering—Part A*.

[22] Heino T. J., Alm J. J., Moritz N., Aro H. T. (2012). Comparison of the osteogenic capacity of minipig and human bone marrow-derived mesenchymal stem cells. *Journal of Orthopaedic Research*.

[23] Castro-Govea Y., Cervantes-Kardasch V. H., Borrego-Soto G. (2012). Human bone morphogenetic protein 2-transduced mesenchymal stem cells improve bone regeneration in a model of mandible distraction surgery. *The Journal of Craniofacial Surgery*.

[24] Luo J., Deng Z.-L., Luo X. (2007). A protocol for rapid generation of recombinant adenoviruses using the AdEasy system. *Nature Protocols*.

[25] Betz O. B., Betz V. M., Nazarian A. (2006). Direct percutaneous gene delivery to enhance healing of segmental bone defects. *The Journal of Bone & Joint Surgery—American Volume*.

[26] Zhu W., Rawlins B. A., Boachie-Adjei O. (2004). Combined bone morphogenetic protein-2 and -7 gene transfer enhances osteoblastic differentiation and spine fusion in a rodent model. *Journal of Bone and Mineral Research*.

[27] Morimoto T., Kaito T., Matsuo Y. (2015). The bone morphogenetic protein-2/7 heterodimer is a stronger inducer of bone regeneration than the individual homodimers in a rat spinal fusion model. *Spine Journal*.

[28] Koh J. T., Zhao Z., Wang Z., Lewis I. S., Krebsbach P. H., Franceschi R. T. (2008). Combinatorial gene therapy with BMP2/7 enhances cranial bone regeneration. *Journal of Dental Research*.

[29] Qing W., Guang-Xing C., Lin G., Liu Y. (2012). The osteogenic study of tissue engineering bone with BMP2 and BMP7 gene-modified rat adipose-derived stem cell. *Journal of Biomedicine & Biotechnology*.

[30] Garrison K. R. K., Shemilt I., Donell S. (2010). Bone morphogenetic protein (BMP) for fracture healing in adults. *The Cochrane Database of Systematic Reviews*.

[31] Barr T., McNamara A. J. A., Sándor G. K. B., Clokie C. M. L., Peel S. A. F. (2010). Comparison of the osteoinductivity of bioimplants containing recombinant human bone morphogenetic proteins 2 (Infuse) and 7 (OP-1). *Oral Surgery, Oral Medicine, Oral Pathology, Oral Radiology and Endodontology*.

[32] Su J., Xu H., Sun J., Gong X., Zhao H. (2013). Dual delivery of BMP-2 and bFGF from a new nano-composite scaffold, loaded with vascular stents for large-size mandibular defect regeneration. *International Journal of Molecular Sciences*.

[33] Jun S.-H., Lee E.-J., Jang T.-S., Kim H.-E., Jang J.-H., Koh Y.-H. (2013). Bone morphogenic protein-2 (BMP-2) loaded hybrid coating on porous hydroxyapatite scaffolds for bone tissue engineering. *Journal of Materials Science: Materials in Medicine*.

[34] Floerkemeier T., Witte F., Nellesen J., Thorey F., Windhagen H., Wellmann M. (2012). Repetitive recombinant human bone morphogenetic protein 2 injections improve the callus microarchitecture and mechanical stiffness in a sheep model of distraction osteogenesis. *Orthopedic Reviews (Pavia)*.

[35] Li J. Z., Li H., Sasaki T. (2003). Osteogenic potential of five different recombinant human bone morphogenetic protein adenoviral vectors in the rat. *Gene Therapy*.

[36] Zhao M., Zhao Z., Koh J.-T., Jin T., Franceschi R. T. (2005). Combinatorial gene therapy for bone regeneration: cooperative interactions between adenovirus vectors expressing bone morphogenetic proteins 2, 4, and 7. *Journal of Cellular Biochemistry*.

[37] Carreira A. C. O., Zambuzzi W. F., Rossi M. C., Filho R. A., Sogayar M. C., Granjeiro J. M. (2015). Bone Morphogenetic Proteins: promising molecules for bone healing, bioengineering, and regenerative medicine. *Vitamins and Hormones*.

[38] Southwood L. L., Kawcak C. E., Hidaka C. (2012). Evaluation of direct in vivo gene transfer in an equine metacarpal IV ostectomy model using an adenoviral vector encoding the bone morphogenetic protein-2 and protein-7 gene. *Veterinary Surgery*.

[39] Dong S.-W., Ying D.-J., Duan X.-J. (2009). Bone regeneration using an acellular extracellular matrix and bone marrow mesenchymal stem cells expressing Cbfa1. *Bioscience, Biotechnology and Biochemistry*.

