# Supplementary material for: Implant Composed of Demineralized Bone and Mesenchymal Stem Cells Genetically Modified with AdBMP2/AdBMP7 for the Regeneration of Bone Fractures in Ovis aries
Source: Stem Cells Int. 2016 Oct 13;2016:7403890. doi: 10.1155/2016/7403890 (PMC5081458; doi:10.1155/2016/7403890)
Supplement: Supplementary file 1 — Table S1. Primers used in this study for qPCR analyses. [file 7403890.f1.pdf]

Table S1. Primers used in this study for qPCR analysis.

Marker	Sequence
CD34	5´-GGTGGCTGATACCGAACTGT-3´
	5´-TCAGCATCTTGGCTGTATGC-3´
CD45	5´-CCACGGGTATTCAGCAAGTT-3´
	5´-CCCAGATCATCCTCCAGAAA-3´
CD116	5´-CGCAATGCAACAGGAGACTA-3´
	5´-GGCTAGATCGAAGCTTGACG-3´
CD73	5´-CTGAGACACCCGGATGAGAT-3´
	5´-ACTGGACCAGGTCAAAGGTG-3´
GAPDH	5´-GTTGTCAGCAATGCCTCCT-3´
	5´-AAGCAGGGATGATGTTTTGG-3´
OC	5´-GCAGCGAGGTGGTG-3´
	5´-CTCCTGGAAGCCGATGTG-3´
Col I	5´-GGTGACAGGAAGTCCCAGAA-3´
	5´-CCATCGTAGGTGACGCTGTA-3’
